# Integrative MicroRNA and Proteomic Approaches Identify Novel Osteoarthritis Genes and Their Collaborative Metabolic and Inflammatory Networks

**DOI:** 10.1371/journal.pone.0003740

**Published:** 2008-11-17

**Authors:** Dimitrios Iliopoulos, Konstantinos N. Malizos, Pagona Oikonomou, Aspasia Tsezou

**Affiliations:** 1 Institute of Biomedical Research and Technology, Larissa, Greece; 2 Department of Orthopaedics, Medical School, University of Thessaly, Larissa, Greece; 3 Laboratory of Cytogenetics & Molecular Genetics, Medical School, University of Thessaly, University Hospital of Larissa, Larissa, Greece; 4 University of Thessaly, Medical School, Department of Biology, Larissa, Greece; Massachusetts Institute of Technology, United States of America

## Abstract

**Background:**

Osteoarthritis is a multifactorial disease characterized by destruction of the articular cartilage due to genetic, mechanical and environmental components affecting more than 100 million individuals all over the world. Despite the high prevalence of the disease, the absence of large-scale molecular studies limits our ability to understand the molecular pathobiology of osteoathritis and identify targets for drug development.

**Methodology/Principal Findings:**

In this study we integrated genetic, bioinformatic and proteomic approaches in order to identify new genes and their collaborative networks involved in osteoarthritis pathogenesis. MicroRNA profiling of patient-derived osteoarthritic cartilage in comparison to normal cartilage, revealed a 16 microRNA osteoarthritis gene signature. Using reverse-phase protein arrays in the same tissues we detected 76 differentially expressed proteins between osteoarthritic and normal chondrocytes. Proteins such as SOX11, FGF23, KLF6, WWOX and GDF15 not implicated previously in the genesis of osteoarthritis were identified. Integration of microRNA and proteomic data with microRNA gene-target prediction algorithms, generated a potential “interactome” network consisting of 11 microRNAs and 58 proteins linked by 414 potential functional associations. Comparison of the molecular and clinical data, revealed specific microRNAs (miR-22, miR-103) and proteins (PPARA, BMP7, IL1B) to be highly correlated with Body Mass Index (BMI). Experimental validation revealed that miR-22 regulated PPARA and BMP7 expression and its inhibition blocked inflammatory and catabolic changes in osteoarthritic chondrocytes.

**Conclusions/Significance:**

Our findings indicate that obesity and inflammation are related to osteoarthritis, a metabolic disease affected by microRNA deregulation. Gene network approaches provide new insights for elucidating the complexity of diseases such as osteoarthritis. The integration of microRNA, proteomic and clinical data provides a detailed picture of how a network state is correlated with disease and furthermore leads to the development of new treatments. This strategy will help to improve the understanding of the pathogenesis of multifactorial diseases such as osteoarthritis and provide possible novel therapeutic targets.

## Introduction

Osteoarthritis is a multifactorial disease characterized by destruction of the articular cartilage due to genetic, mechanical and environmental components [1], affecting more than 20 million people in the US [2]. Despite its high prevalence there are few studies concerning the molecular pathobiology and the involvement of genetic factors in the pathogenesis of osteoarthritis [3], [4]. Several clinical studies have implicated the causative role of obesity in osteoarthritis development [5], [6], however there are few molecular studies correlating metabolism with osteoarthritis [7], [8]. Achieving a deeper understanding of osteoarthritis molecular mechanisms requires global strategies aimed at modelling the functional interrelationships between genes as complex interdependent networks.

Lately, it has become evident that genetic alterations in non-coding genes can also contribute to the pathogenesis of human disease [9]. A new class of small non-coding RNAs, named microRNAs, regulate gene expression by inhibition of translation or mRNA cleavage [10]. MicroRNAs have been implicated in important cellular processes such as lipid metabolism [11], apoptosis [12], differentiation [13] and organ development [14]. Furthermore microRNAs expression signatures have been associated with well-defined clinicopathological features and disease outcome [15]. It is known that microRNAs exert their biological functions through suppression of their target genes. Several bioinformatic algorithms have been constructed in order to predict microRNA gene targets. Most of these algorithms search for sequence complementarity between the microRNA and the 3′ UTR of the gene target. These algorithms predict hundreds of potential gene targets, which can not all be experimentally validated. Previous studies have tried to identify microRNA gene targets using cDNA microarray data [16]. However, it has been shown that a microRNA (miR-10b) regulates gene expression only at the protein level, while mRNA levels were not affected [17]. In addition, very recently Selbach et al, showed that a microRNA can repress the production of hundred of proteins [18]. Therefore, it becomes evident that proteomic data are needed in order to accurately detect microRNA gene targets. Up to now there are few studies trying to characterize the cartilage proteome. More specifically, recently Vincourt et al, performed a detailed two dimensional electrophoresis-based proteomic analysis of articular cartilage [19]. In addition Wu et al. performed a comparative proteomic analysis of cartilage from healthy donors and osteoarthritis patients, however the number of samples used was very small [20].

For all above reasons, we undertook to associate specific microRNAs and proteins with the development of osteoarthritis and clinicopathological parameters, in order to identify new signalling pathways involved in its pathogenesis. Here, we report a novel approach of studying multi-aetiological diseases and identifying new genes involved in the pathogenesis of a complex disease. Integration of microRNA microarray and proteomic analysis data together with computational approaches, such as microRNA gene target prediction algorithms and gene network construction, revealed the role of microRNAs in cartilage destruction and linked inflammatory and metabolic gene networks with cartilage homeostasis.

## Materials and Methods

### Cartilage tissue samples

Articular cartilage samples were obtained from femoral heads, femoral condyles and tibial plateaus of patients with primary osteoarthritis undergoing hip or knee replacement surgery at the Orthopaedics Department of University Hospital of Larissa. A total of 33 patients were included in this study (twenty eight females and five males; mean age 68.91±6.97 years, range 57–83; mean Body Mass Index (BMI) 30.51±5.23, range 22.67–43.96) who had undergone total knee replacement surgery. Each sample was categorized according to its gross morphology, as severely damaged and was taken from the main defective area of maximal load. Macroscopic findings were validated by histological studies performed on 5 mm serial sections of cartilage samples and graded using the Mankin score. Specimens with osteoarthritis had Mankin score 10–14. Normal cartilage was obtained from small free cartilaginous fragments from ten individuals (six females and four males; mean age 61.70±18.17, range 27–78; mean BMI 23.80±4.34, range 19.03–35.06) with 0 Mankin score, undergoing fracture repair surgery, with no history of joint disease. Both patients and healthy individual's cartilage samples were obtained upon individuals' verbal informed consent. The method of obtaining verbal approval by all individuals was approved by Institutional Review Board of the University Hospital of Larissa. Also the protocol was approved by the local ethics committee of University Hospital of Larissa.

### Detection of microRNA expression

Expression levels of 365 microRNAs were evaluated with TaqMan microRNA microarray assays as previously described [21]. Validation of these results was performed using the mirVana qRT–PCR miRNA Detection Kit and qRT–PCR Primer Sets, according to the manufacturer's instructions (Ambion Inc, TX, USA). The U6 small nuclear RNA was used as an internal control.

### MicroRNA Northern Blot Analysis

For microRNA Northern Blot Analysis, 10ug of RNA were separated on 12% denaturating polyacrylamide gels and transferred to GeneScreen Plus membrane (PerkinElmer, Waltham). MiRCURY LNA Probes for miR-483 and miR-22 (Exiqon, Denmark) were end-labeled with T4 polynucleotide kinase. Prehybridization of the filters was carried out in 50% formamide, 0.5% SDS, 5· SSPE, 5·Denhardt's solution and 20 mg/ml sheared, denatured salmon sperm DNA. Hybridizations were performed in the same solution at 42°C. The labeled probes were heated for 1 min at 95°C before addition to the filters in the prehybridization solution. After hybridization, the membranes were washed in 0.1 SSC, 0.1% SDS at 42°C twice for 10 min.

### Reverse-Phase protein microarray analysis

Chondrocyte cell lysates were boiled for 5 min and were loaded into 384-well plates in serial dilutions (neat, 1∶2, 1∶4, 1∶8, and 1∶16) with negative control wells containing only lysis buffer. These samples were printed in duplicate onto nitrocellulose-coated glass slides (Schleicher & Schuell Bioscience, Keene, NH) using a ring-and-pin robotic arrayer (GMS 417, Affymetrix, Santa Clara, CA). The arrays were stained as previously described [22] on an autostainer (DAKO, Carpinteria, CA) using a biotinyl-linked catalyzed signal amplification system (DAKO). Specificity of each antibody was tested by western blot analysis.

### Statistical analysis

All calculations were performed on a Microsoft computer, using the SPSS software (version 12.0). Correlation of between microRNA and protein expression levels with BMI was identified by correlation coefficients, calculated by Pearson rank correlation (*r*) and Spearman rank correlation. Statistical methods regarding the proteomic analysis are described analytically in the suppl. Methods section. Construction and statistical significance of gene networks was performed by Ingenuity pathway analysis. Statistical significant networks were considered those with p value higher than 10^−5^. In addition clustering of the protein data in functional groups was performed using DAVID NIH Bioinformatics Database with a p value cut-off of 10^−5^. Quantification of western blots was performed by standard densitometric analysis. All transfection experiments were performed in triplicate and the results were compared by student's t-test analysis.

### Additional methods

Detailed experimental methods are described in the supplemental methods section (**Methods S1**).

## Results

### MicroRNA gene signature of osteoarthritis

**Figure 1:**
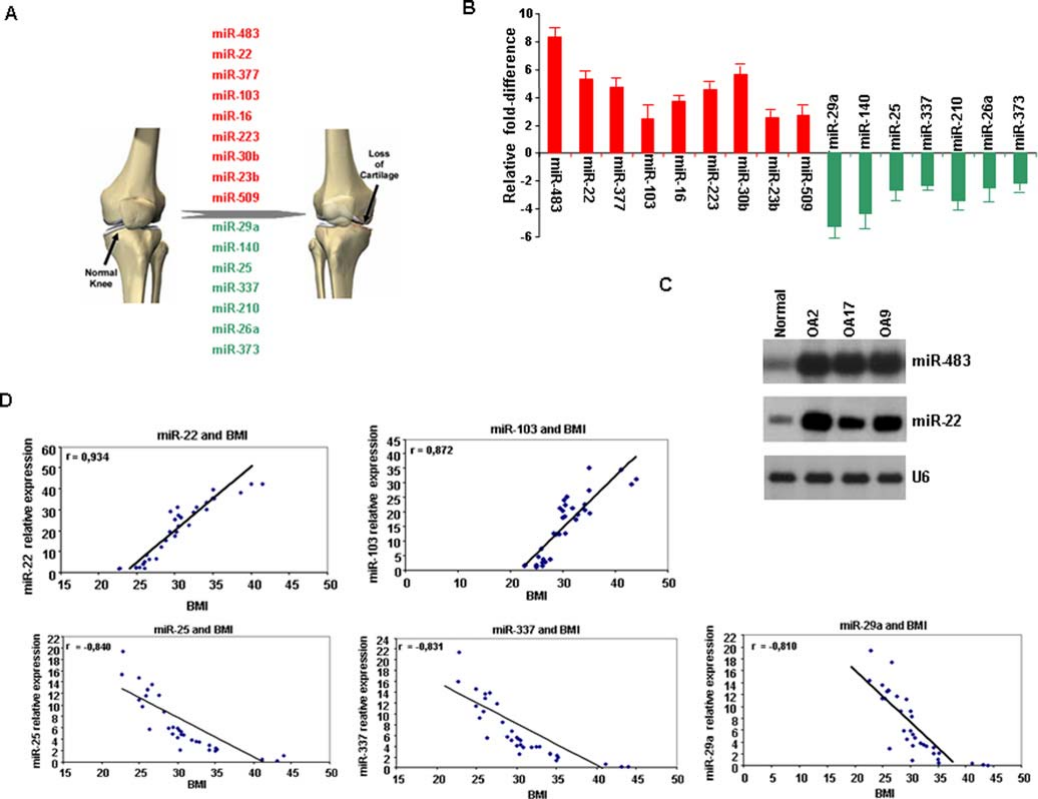
MicroRNA gene signature in osteoarthritis and correlation with clinicopathological parameters. (A) Up-regulated (red color) and down-regulated (green color) microRNAs in 33 osteoarthritic and 10 normal cartilage samples assayed by TaqMan microRNAs assays. Included microRNAs were more than 2-fold deregulated. (B) Validation of previous results using Real-time SYBR Green microRNA detection assay. (C) Northern blot validation of microRNA microarray data. Representative examples of miR-483 and miR-22 expression in normal and osteoarthritic cartilage tissues (OA2, OA17, OA9). (D) MicroRNAs correlated with BMI (Body Mass Index) analyzed by SSPS version 12.0 statistical program.

To identify microRNAs involved in osteoarthritis, we tested the expression of 365 microRNAs in articular cartilage obtained from patients with osteoarthritis undergoing knee replacement surgery and from normal individuals with no history of joint disease. We identified 16 microRNAs differentially expressed in osteoarthritic compared to normal cartilage (**Figure 1A**). Specifically we detected nine up-regulated and seven down-regulated microRNAs in osteoarthritic cartilage compared to normal (**Table S1**). Real-time PCR and Northern blot analysis (**Figure 1B, C** **;** **Table S2**) validated that this 16 microRNA gene signature was able to distinguish osteoarthritic from normal chondrocytes.

### microRNA expression correlates with BMI

Clinical characteristics of the patients and normal individuals (**Table 1**) allowed us to study potential correlations between microRNAs expression and clinicopathological parameters. Very interestingly, we found five microRNAs to be statistically correlated with body mass index (BMI) (**Figure 1C**). miR-22 and miR-103 expression was positively correlated with BMI, while miR-25, miR-337 and miR-29a expression was inversely correlated, pointing towards the potential role of microRNAs in lipid metabolism and osteoarthritis pathogenesis.

**Table 1:** Clinicopathological characteristics of osteoarthritis patients and normal individuals

	OA	Normal
**Characteristic**		
**Female Sex – no (%)**	28 (84.8%)	6 (60%)
**Age at diagnosis -yr**		
Median	68.91±6.97	61.70±18.17
Range	57–83	27–78
**Body Mass Index (BMI)**		
Median	30.51±5.23	23.80±4.34
Range	22.67–43.96	19.03–35.06
Normal	4	6
Overweight	11	2
Obese	18	2
**Kellgrene-Lawrence**		
Median	3.72±0.51	0
Range	2–4	0

### Proteomic analysis of articular cartilage

**Figure 2:**
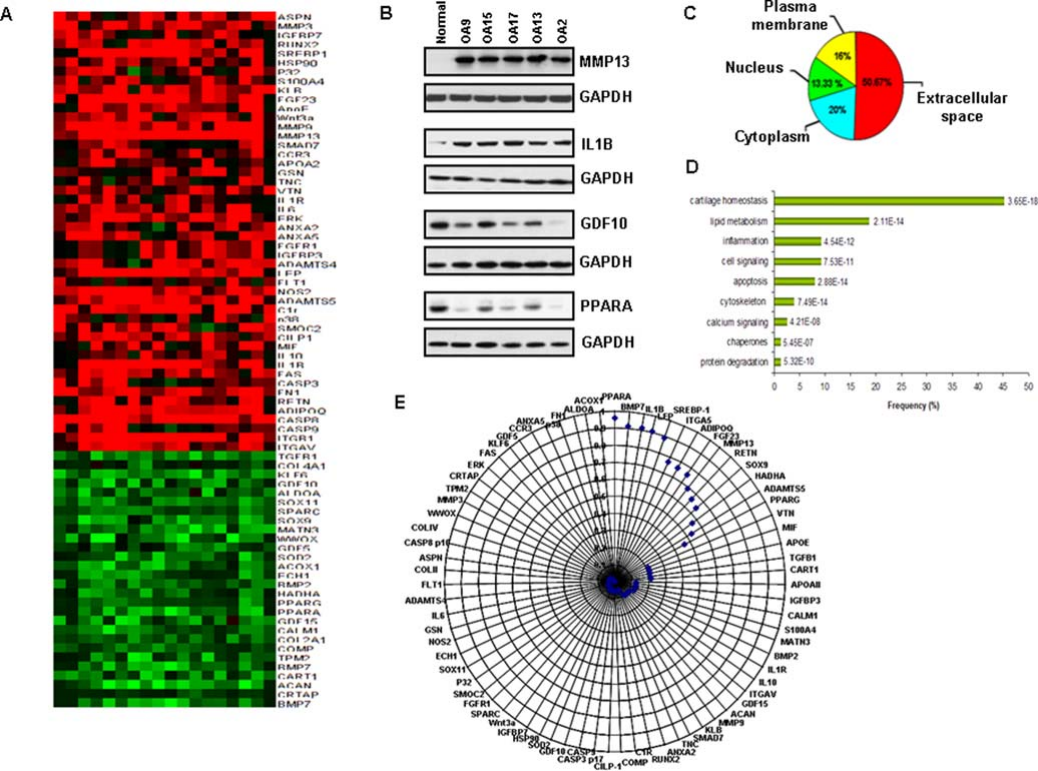
Reverse phase protein arrays in osteoarthritic and normal cartilage tissues. (A) Differentially expressed proteins between osteoarthritic and normal chondrocytes. Up-regulated are shown with red color, while down-regulated with green color. (B) Representative western blot analysis in protein extracts from five osteoarthritic tissues in comparison with normal cartilage. (C) Sub-cellular localization of differentially expressed proteins. (D) Functional clustering analysis of differentially expressed proteins (using DAVID NIH Bioinformatic Database). (E) Correlation coefficient wheel between protein expression levels of differentially expressed proteins in osteoarthritic vs normal chondrocytes and body mass index (BMI). We identified 3 protein groups, which showed statistically significant correlations between protein expression and BMI, according to the coefficient correlation index (r^2^). More specifically, the first group with the highest correlation (r^2^>0.900) consisted of PPARA, BMP7, IL1B, LEP (leptin) and SREBP1 proteins. The second group (0.600>r^2^>0.900) consisted of ITGA5, ADIPOQ (adiponectin), FGF23, MMP13, RETN (resistin) and SOX9. The third group (0.400>r^2^>0.600) consisted of 3 proteins (HADHA, ADAMTS5, PPARG) which had low degree of correlation with BMI. The rest of the proteins were not correlated with BMI.

In order to identify deregulated proteins in osteoarthritic chondrocytes and study in detail whether obesity and osteoarthritis are correlated at a molecular level, we performed proteomic analysis in the same articular cartilage samples that we performed microRNA expression analysis. Specifically reverse-phase protein arrays were constructed [23] (**Figure S1A**) and probed with antibodies to 214 proteins expressed in articular cartilage (**Table S3**). All antibodies were tested for their quality and specificity by Western blot analysis (**Figure S1B**) Arrays were scanned and dilution curves were used to quantify relative protein expression (**Figure S1C**). We detected that 76 proteins, (48 up-regulated and 28 down-regulated) were differentially expressed between osteoarthritic and normal chondrocytes (**Figure 2A**). These results were validated by western blot analysis (**Figure 2B**). We were able to identify for the first time deregulated proteins in osteoarthritic chondrocytes that were implicated in inflammatory and lipid metabolism pathways. More specifically, we detected up-regulation of proteins involved in inflammatory pathways such as IL1B, IL6 and CCR3, while proteins (PPARA, PPARG, ACOX1) involved in lipid metabolism mechanisms, were found highly down-regulated in osteoarthritic chondrocytes (**Figure 2A, B**). In addition we detected novel proteins that were deregulated in osteoarthritis, such as SOX11, FGF23, KLF6, WWOX and GDF15.

Around half of the deregulated proteins in osteoarthritic chondrocytes were located in the extracellular space (50.67%), while the rest had cytoplasmic (20%), plasma membrane (16%) and nuclear (13.33%) localization (**Figure 2C**). Functional clustering analysis categorized the differentially expressed proteins in nine statistically significant pathways. Specifically, 45.33% of the deregulated proteins were involved in cartilage homeostasis pathways (**Figure 2D**), while 18.67% and 9.33% were involved in lipid metabolism and inflammation pathways, respectively.

### Metabolism-related proteins correlate with BMI

Furthermore, we tried to correlate the expression levels of differentially expressed proteins in osteoarthritic cartilage with clinicopathological parameters, such as Body Mass Index. A recent study suggested that BMI is a significant risk factor for knee osteoarthritis leading to arthroplasty, speculating that biomechanics and metabolic factors associated with adipose tissue contribute to this phenotype [5]. We identified that PPARA, BMP7, IL1B, LEP, ITGA5 and SREBP1 protein levels in osteoarthritic chondrocytes were highly correlated with BMI (**Figure 2E**), suggesting the potential role of metabolic-related proteins in the development of osteoarthritis.

### Detection of microRNA gene targets

Since microRNAs exert their biological functions through suppression of target genes, it is important to identify microRNA-target pairs. As the available bioinformatic algorithms predict hundreds of microRNA-gene target pairs, it is evident that experimental data are needed for verification of these pairs. However, as it is known that several microRNAs target gene expression only at the protein and not at the mRNA level, the integration of microRNA along with protein data sets could be considered more effective for microRNA-gene target verification. Recently Huang *et al.,* used microRNA with cDNA expression profiling data to identify human microRNA targets using Bayesian data analysis algorithm [16]. In our study, we identified microRNA-gene target pairs by matching microRNA and protein data. Subsequently, we filtered these data through three different selection criteria (**Figure S2**) and revealed 17 microRNA-gene target pairs implicated in osteoarthritis pathogenesis (**Table S4**). More specifically, we found microRNA-gene target pairs potentially involved in cartilage homeostasis and structure (miR-377-CART1, miR-140-ADAMTS5, miR-483-ACAN, miR-23b-CRTAP, miR-16-TPM2, miR-223-GDF5, miR-509-SOX9, miR-26a-ASPN), in biomechanic pathways (miR-25-ITGA5), in apoptotic mechanisms (miR-373-CASP6, miR-210-CASP10) and in lipid metabolism pathways (miR-22-PPARA, miR-22-BMP7, miR-103-ACOX1, miR-337-RETN, miR-29a-LEP). Several of these target genes such as ADAMTS5, GDF5 and LEP have been previously correlated with osteoarthritis [3], [24], [25].

### Gene networks in osteoarthritis

**Figure 3:**
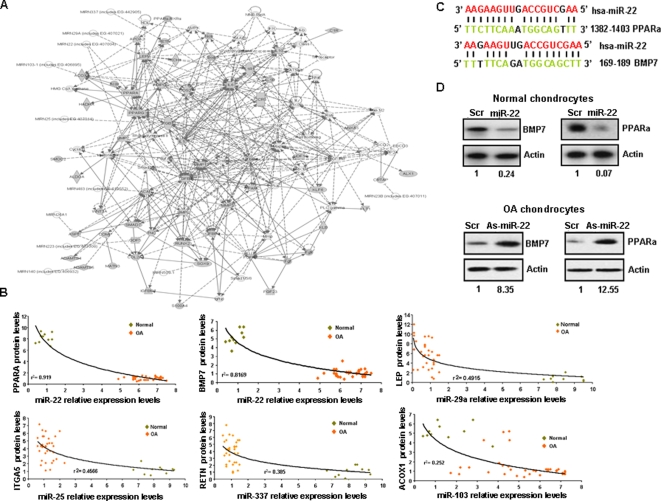
Interactome network in osteoarthritis. (A) Construction of an interactome network (*p* = 10-^49^) by integrating microRNA and proteomic data using Ingenuity Pathway Analysis (IPA) (more information in suppl. methods). The *p* value indicates the likelihood of focus genes to belong to a network versus those obtained by chance. Around half of the microRNAs (mir-337, miR-29a, miR-22, miR-103-1, miR-25) regulate genes involved in the metabolic pathways. (B) Correlation coefficients between microRNA expression levels and their gene targets protein levels in osteoarthritic and normal chondrocytes. (C) Predicted duplex formation between PPARA and BMP7 3′UTR with miR-22. (D) BMP7 and PPARA protein levels after miR-22 or inhibitor of miR-22 (as-miR-22) treatment (50 nM) for 48 h in normal and osteoarthritic chondrocytes, respectively.

It is becoming increasingly clear that most proteins interact in complex cellular networks, the properties of which might be altered in osteoarthritic compared to normal chondrocytes. Therefore, global strategies aimed at modeling the functional interrelationships between microRNA and proteins, as complex interdependent networks, are required. We integrated microRNA and protein data sets in order to generate a model of macromolecular network that is perturbed in osteoarthritis using Ingenuity program analysis [26]. The resulting network contained 11 microRNAs, 58 proteins and 414 potential functional associations (**Figure 3A**). We were able to detect three sub-networks representing key functional units that make up the co-expression network. A metabolism-related, an inflammation and a cartilage homeostasis sub-network were found to be interrelated contributing all together to cartilage destruction and osteoarthritis development (**Figure S3**).

### miR-22 correlates with PPARA and BMP7 protein expression

The functional significance of our predicted “interactome” network was tested by experimental validation. Our finding that specific microRNAs and proteins were related to BMI, focused our interest in identifying functional microRNA-gene target pairs relating obesity with osteoarthritis pathogenesis mechanisms. Epidemiological studies have shown that the risk for knee osteoarthritis is increased by 36% for every 2 units of BMI (5 kg) of weight gain [27]. At first we tried to identify which microRNA-gene target interactions have biological significance (inverse correlation in expression levels) in our network. According to our previous combined *in silico* and expression data analysis we identified 5 microRNAs (miR-22, miR-103, miR-337, miR-25, miR-29a) and their 6 targets (PPARA, BMP7, ACOX1, RETN, ITGA5, LEP) that were highly correlated with BMI (**Figure 1C** **,** **Figure 2E**). Correlation of microRNA-gene target expression levels revealed that miR-22 was highly inversely correlated with PPARA (r^2^ = 0.919) and BMP7 (r^2^ = 0.816). (**Figure 3B**), while we detected low correlation between miR-29a with LEP (r^2^ = 0.491), miR-25 and ITGA5 (r^2^ = 0.456), miR-337 and RETN (r^2^ = 0.385) and miR-103 and ACOX1 (r^2^ = 0.252). The above results suggested the potential functional relationship between miR-22, PPARA and BMP7.

### miR-22 regulates PPARA and BMP7 in normal and osteoarthritic chondrocytes

In order to detect whether PPARA and BMP7 were direct targets of miR-22, we performed luciferase assay. We found that BMP7-encoded mRNA contains a 3′UTR element that is partially complementary to miR-22 (**Figure 3C**) and luciferase assay showed that BMP-7 is a direct target of miR-22 (69% reduction, p<0.001, **Figure S4A**). Furthermore, miR-22 overexpression in chondrocytes reduced the activity of a luciferase reporter gene fused to the PPARA 3′UTR (52% reduction, p<0.001, **Figure S4B**). Evaluation of BMP7 and PPARA mRNA expression levels after miR-22 expression revealed that only BMP7 mRNA levels were significantly down-regulated (p<0.001), while PPARA were not (p = 0.492) (**Figure S5**). Western blot analysis revealed that miR-22 regulates both PPARA and BMP7 protein expression levels in normal and osteoarthritic chondrocytes (**Figure 3D**). Overexpression of miR-22 inhibited BMP-7 (76%) and PPARA (93%) protein expression in normal chondrocytes. Subsequently, inhibition of miR-22 in osteoarthritic chondrocytes by antisense miR-22 treatment, highly up-regulated BMP-7 (8.35 fold) and PPARA (12.55 fold) expression, suggesting that miR-22 is a strong regulator of BMP-7 and PPARA proteins. All above data suggest that miR-22 regulates BMP-7 at the mRNA level and PPARA at the protein level, revealing thus the advantage of using proteomic instead of cDNA microarray data for detecting microRNA gene targets.

### Metabolic, inflammatory and cartilage homeostasis networks are inter-related

**Figure 4:**
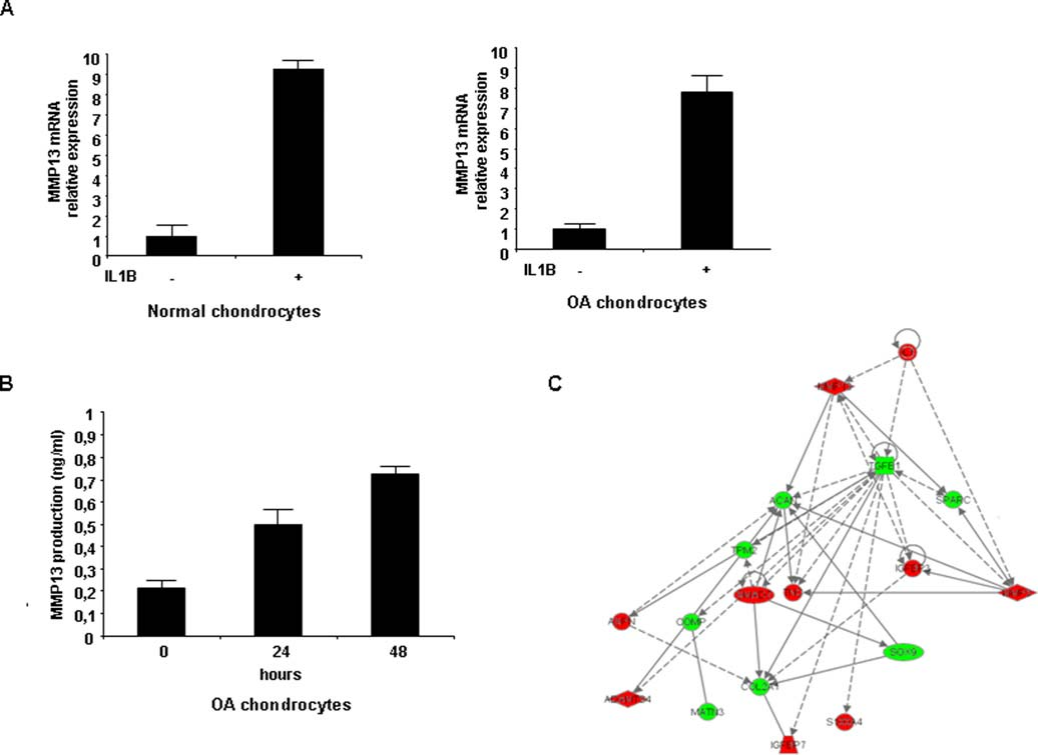
IL1B regulates important components of cartilage homeostasis network. (A) Treatment with IL1B (10 ng/ml) induces MMP-13 mRNA levels assessed by Real-time PCR analysis in normal and osteoarthritic chondrocytes. (B) ELISA assay detecting MMP-13 levels after IL-1b treatment of osteoarthritic chondrocytes. (C) Pertubation of IL1B affects important gene network components in normal chondrocytes. Treatment of normal chondrocytes with IL1B (10ng/ml) for 48 h affects the protein expression of cartilage structure related genes (red color shows up-regulation while green color down-regulation of protein expression). This experiment was performed in quadruplicate. Specifically there is activation of metalloproteinases 3 and 13 (MMP3, MMP13) and aggrecanases (ADAMTS4, ADAMTS5) leading to down-regulation of the cartilage structural proteins (ACAN, SPARC, COMP, TPM2, MATN3, COL2A1). In addition asporin (ASPN) is up-regulated which has been shown to inhibit TGF-beta and aggrecan synthesis (look ref 11). Furthermore SOX9, an important trascription factor implicated in chondrogenesis is highly down-regulated.

Subsequently, we proceeded by investigating how these miR-22 target gene pairs are correlated with the rest of the proteins present in the “interactome” network. Specifically, PPARA belongs to the metabolism sub-network, which is connected with the inflammation sub-network through IL1B. A recent report suggested that PPARA is a receptor involved in inflammatory processes [28] and was recently found down-regulated in osteoarthritic cartilage [29]. In our study, the inflammatory sub-network having IL1B and IL6 as central nodes is connected with MMP13, which is central node of the cartilage structure sub-network. To test this hypothesis predicted by the gene network of IL1B-MMP13 interaction, we treated normal and osteoarthritic chondrocytes with IL1B and examined MMP13 expression. We found that IL1B up-regulated MMP13 expression both at mRNA and protein levels (**Figure 4A, B**), verifying the correlation that we had previously described between IL1B and MMP13 expression levels in clinical samples [30]. In order to understand how IL1B affects not just MMP13 but cartilage homeostasis pathways, we over-expressed IL1B in normal chondrocytes and monitored the expression of the proteins involved in the cartilage network. IL1B over-expression pertubated the cartilage homeostasis sub-network by activation of metalloproteinases and aggrecanases and down-regulation of cartilage structural proteins. (**Figure 4C**), connecting thus inflammation and cartilage homeostasis sub-networks with osteoarthritis development.

### PPARA and BMP7 regulate IL1B and MMP13 expression in chondrocytes

**Figure 5:**
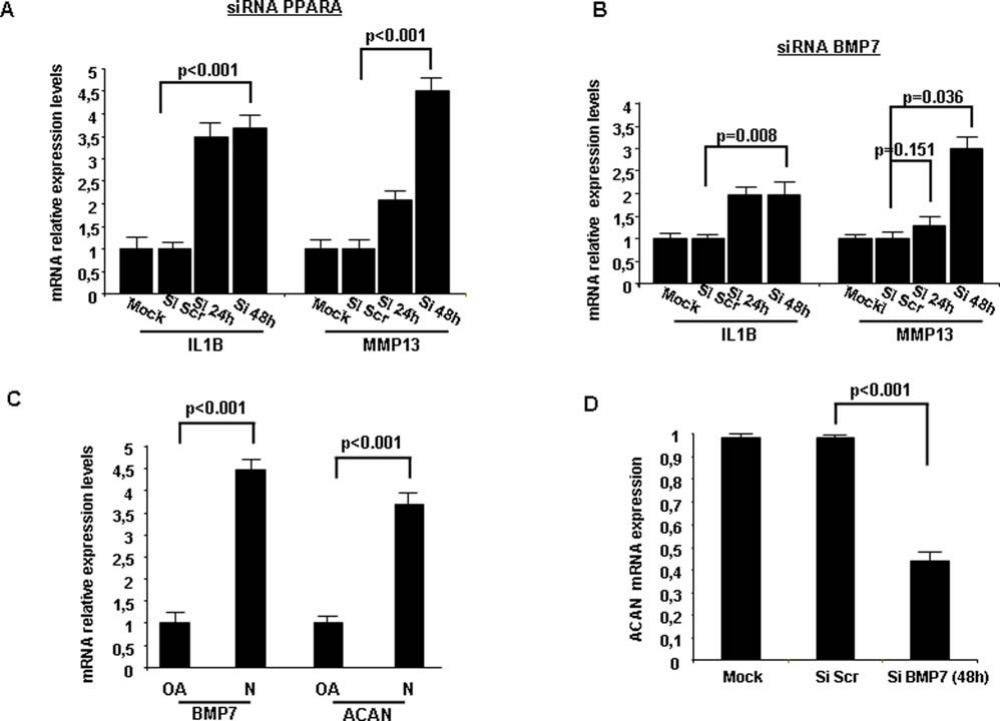
PPARA and BMP7 signaling pathways in chondrocytes. (A) Assessment of IL1B and MMP13 mRNA levels after down-regulation of PPARA, 24 and 48 h after siRNA liposomal treatment into chondrocytes. (B) IL1B and MMP13 expression 24 and 48 h after BMP7 siRNA treatment. (C) Evaluation and correlation of BMP7 and ACAN (aggrecan) mRNA levels in normal and osteoarthritic chondrocytes assessed by real-time PCR analysis. (D) Aggrecan expression levels 48 h after BMP7 inhibition of expression using siRNA transferred by liposomes into normal chondrocytes. All experiments have been performed in triplicate.

In order to delineate the PPARA-IL1B-MMP13 potential pathway we followed an RNA interference strategy. More specifically, siRNA inhibition of PPARA increased IL1B (2.5 fold) and MMP13 (3.5 fold) expression levels (**Figure 5A**). IL1B was found to be highly induced 24 h after siRNA PPARA treatment, while MMP13 was highly induced 48 h after siRNA treatment, suggesting a sequential activation of MMP13 by IL1B.

The second target of miR-22, BMP7, is frequently down-regulated in osteoarthritic cartilage, while BMP7 overexpression induces cartilage formation *in vitro* and *in vivo* [31]. It has been shown that IL1B and ACAN (aggrecan) [32] levels are regulated by BMP7 in osteoarthritic cartilage. These correlations were present in the cartilage homeostasis sub-network and in addition we found that siRNA against BMP7 resulted in increased IL1B and MMP13 (2 fold) expression in normal chondrocytes (**Figure 5B**). Furthermore, we detected high correlation between BMP7 and ACAN mRNA levels (**Figure 5C**) and found that BMP7 siRNA down-regulation blocked effectively ACAN expression (**Figure 5D**).

### miR-22 blocks MMP13 activity and inhibits cartilage destruction

**Figure 6:**
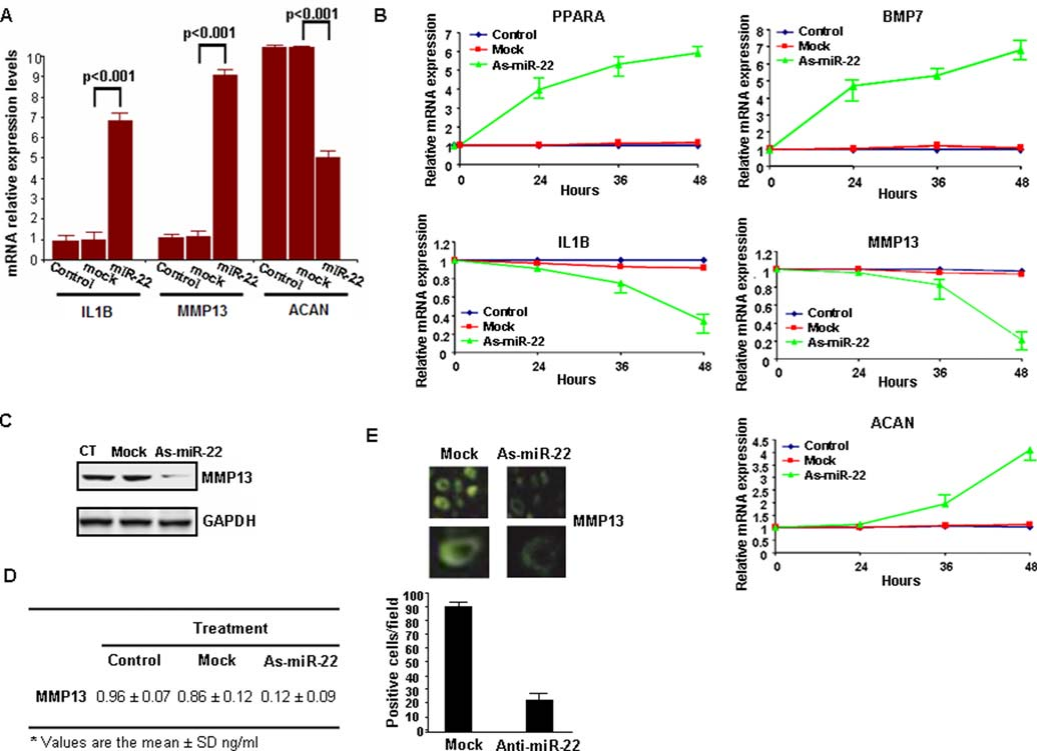
miR-22 regulates PPARA and BMP7 signaling pathways in human chondrocytes. (A) Evaluation by real-time PCR analysis of IL1B, MMP13 and ACAN mRNA expression levels 48 h after miR-22 (50 nM) liposomal transfection in normal chondrocytes. Real-time PCR analysis has been performed in triplicate. (B) Assessment of IL1B, MMP13 and ACAN mRNA levels after antisense-miR-22 transfection in osteoarthritic chondrocytes. As-miR-22 treatment affects very early (24 h) PPARA and BMP7 mRNA expression, while IL1B, MMP13 and ACAN expression is affected later (36–48 h) suggesting that there are secondary effects. Real-time PCR analysis has been performed in triplicate. (C, D) Western blot analysis and ELISA assay for MMP13 expression after as-miR-22 overexpression. In ELISA assay each sample has been loaded in quadruplicate and the assay has been performed in triplicate (average and standard deviation is shown). (E) MMP13 expression evaluated by immunofluorescence analysis of osteoarthritic chondrocytes after as-miR-22 liposomal transfection. In the bar graph is shown the average of MMP-13 expressing (green fluorescent) cells detected in 20 different fields in the microscope.

Overexpression of miR-22 in normal chondrocytes resulted in increased IL1B (5.8 fold) and MMP13 (8.1 fold) expression and decreased aggrecan (4.9 fold) expression (**Figure 6A**). These results point towards the implication of the combinatory effect in MMP13 up-regulation (8.1 fold instead of 3.5 and 2 fold) by miR-22 overexpression through PPARA and BMP7. Additionally, inhibition of miR-22 in osteoarthritic chondrocytes up-regulated PPARA (4.9 fold) and BMP7 (5.8 fold) expression, blocked the inflammatory process, through inhibition of IL1B (7.6 fold), inhibited catabolic changes such as MMP13 expression (7.9 fold) and activated the cartilage repair protein aggrecan (3.1 fold) (**Figure 6B**), suggesting the therapeutic potential of microRNA inhibition in osteoarthritis. Furthermore, Western blot, ELISA and immunofluorescence experiments revealed a high decrease of MMP13 expression in osteoarthritic chondrocytes after miR-22 inhibition (**Figure 6 C–E**). MMP13 is one of the major pathophysiological mediators of cartilage destruction, through degradation of type II collagen in osteoarthritis [33] and therefore its down-regulation is of great clinical importance.

## Discussion

We detected, for the first time to our knowledge, a 16 microRNA gene signature differentially expressed in osteoarthritis. The biological significance of microRNAs is determined by their gene targets which currently can be predicted computationally by prediction algorithms. In order to have experimental data for identification of microRNA gene targets, we performed proteomic analysis, using reverse-phase protein arrays, which revealed a 76 protein signature differentially expressed in osteoarthritis. This technology platform has been designed for quantitative multiplexed analysis of cellular proteins from a limited amount of sample [34], offering thus a major advantage for protein quantification levels from tissues such as cartilage, where the material is frequently limited.

Proteomic analysis was validated by detecting differentially expressed genes such as GDF5, that have been identified as osteoarthritis susceptibility genes by gene association studies [3]. Up to date most osteoarthritis molecular studies focus their interest in cartilage homeostasis mechanisms. However our global approach identified proteins related with osteoarthritis pathobiology, that are involved in lipid metabolism and inflammatory pathways, providing a new source of protein expression data that need to be explored in greater detail. For example, we identified novel proteins such as SOX11, CCR3, WWOX to be differentially expressed in osteoarthritic cartilage. Sock et al, have shown that SOX11 is an important transcription factor related with skeletal malformations [35]. Similarly, recently Aqeilan et al showed that WWOX -/- mice develop metabolic bone disease [36]. In addition CCR3 has been found up-regulated in adipose tissue from obese individuals [37]. All these studies suggest that these proteins are directly or indirectly related with osteoarthritis and their differential expression observed in our study in osteoarthritic chondrocytes supports the idea that these proteins are involved in cartilage destruction pathways. However, additional molecular studies are needed to clarify the role and importance of these proteins in osteoarthritis development.

Comparison of proteomic and clinical data revealed metabolism-related proteins differentially expressed in osteoarthritic chondrocytes to be correlated with BMI. It has been described that mechanoreceptors are activated in knee chondrocytes due to excess of weight contributing to cartilage destruction. Very interestingly, our proteomic analysis revealed that ITGA5 mechanoreceptor protein was highly up-regulated in osteoarthritic chondrocytes and was correlated with BMI. Previous studies have described integrin-dependent signalling cascades in chondrocyte mechanotransduction. Furthermore Chowdhury et al., showed an integrin-mediated mechanotransduction in IL1B-stimulated chondrocytes, suggesting the relationship between cartilage structure and inflammatory pathways [38].

The present work has led to the identification of an “interactome” network involved in the pathogenesis of osteoarthritis. We have shown that integration of microRNA, protein expression and clinical data can be used to generate a network of potential functional associations with osteoarthritis. We were able to validate experimentally the interplay between metabolism genes, inflammatory molecules and cartilage homeostasis enzymes through microRNA mechanism of action. Specifically we found regulation of IL1B and MMP13 by PPARA and BMP7 through miR-22. Consistent with our studies, Watters *et al*, showed that PPARA expression is reduced in a STR/Ort osteoarthritis mouse model [29]. IL1B is a central node in our network and perturbation of its expression (over-expression) contributes to cartilage destruction. Our molecular data are consistent with several clinical studies implicating the role of obesity and inflammation in cartilage destruction. Several clinical studies have suggested the effect of obesity in the development of osteoarthritis [6], [7], however there are no extensive studies correlating lipid metabolism with osteoarthritis at the molecular level. Specifically, Lohmander et al., in a large population study revealed that body mass is a significant risk factor for osteoarthritis leading to arthroplasty [5]. In addition Marks proposed that a high body mass is present in most adults with osteoarthritis [39].

Gene network approaches provide new insights for elucidating the complexity of diseases such as osteoarthritis. Several genes consist the sub-networks that are interconnected creating large gene networks. Alterations in gene expression that are able to perturb a network have a causal relationship with disease [40]. The integration of gene expression profiling and clinical data provides a detailed picture of how a network state is correlated with disease and furthermore leads to the development of new treatments that target the gene network as opposed to current therapeutic approaches focused on targeting one specific gene only. This strategy will help to improve the understanding of the pathogenesis of multifactorial diseases such as osteoarthritis and provide possible novel therapeutic targets.

## Supporting Information

Methods S1 (0.04 MB DOC)

Table S1 (0.07 MB PPT)

Table S2 (0.07 MB PPT)

Table S3 (0.13 MB PPT)

Table S4 (0.07 MB PPT)

Figure S1 (0.16 MB PPT)

Figure S2 (0.12 MB PPT)

Figure S3 (0.68 MB PPT)

Figure S4 (0.14 MB PPT)

Figure S5 (0.14 MB PPT)
