# Supplementary material for: Integrative MicroRNA and Proteomic Approaches Identify Novel Osteoarthritis Genes and Their Collaborative Metabolic and Inflammatory Networks
Source: PLoS One. 2008 Nov 17;3(11):e3740. doi: 10.1371/journal.pone.0003740 (PMC2582945; doi:10.1371/journal.pone.0003740)
Supplement: Methods S1 — (0.04 MB DOC) [file pone.0003740.s001.doc]

**SYPPLEMENTARY METHODS**

**Cartilage tissue samples.**

Detailed clinical data were obtained from patients and controls regarding grade of physical activity, BMI, presence of pain, as well as stiffness and swelling of the joint. Categories regarding overweight and obesity were constructed based on a graded classification that applies to both men and women proposed by World Health Organization: normal BMI <25 kg/m^2^, overweight BMI 25.0 – 29.9 kg/m^2^ and obese BMI ≥30.0 kg/m^2^. Radiographs were obtained before surgery and graded according to the Kellgren - Lawrence system. According to this system 26 patients had score 4, 7 patients had score 3 and 1 patient had score 2. The radiographs were assessed by two independent observers who were blinded to all data of the individuals. Patients with rheumatoid arthritis and other autoimmune disease as well as chondrodysplasias, infection-induced OA and posttraumatic OA were excluded from the study. The study protocol conformed to the ethical guidelines of the 1975 Declaration of Helsinki as reflected in a priori approval by the Local Ethical Committee of the University Hospital of Larissa.

**Isolation of RNA from human cartilage and cultured chondrocytes.** Fresh tissue, within 1 h from surgery, was dissected, and total cellular RNA was extracted using Trizol reagent (Invitrogen, Life Technologies, Paisley, UK). Cultured chondrocytes were trypsinized, collected and centrifuged for 10 min 1700 rpm and total cellular RNA was extracted using Trizol reagent. Preservation of 28S and 18S ribosomal RNA (rRNA) species was used to assess RNA integrity. All the samples included the study were with prominent 28S and 18S rRNA components. The yield was quantified spectrophotometrically.

**Reverse-Phase protein microarray analysis.** Chondrocytes were lysed in 15 ml of lysing buffer containing 1 : 1 mixture of 26SDS electrophoresis buffer (125 mM Tris pH 6.8, 4% SDS, 10% glycerol, 2% b-mercaptoethanol) and Tissue Protein Extraction Reagent (TPER, Pierce, Rockford, IL, USA) for 2 h at 70oC. The samples were printed onto nitrocellulose-coated glass slides (Schleicher & Schuell Bioscience, Keene, NH) using a ring-and-pin robotic arrayer (GMS 417, Affymetrix, Santa Clara, CA). Stained slides were scanned on a UMAX scanner with Adobe PhotoShop 7.0 at a resolution of 600 dpi for analyses. Total protein content of each array spot was detected using Sypro Ruby Protein Blot Stain (Molecular Probes, Eugene, OR) and a fluorescence imaging system (Alpha Innotech, San Leandro, CA). Mean pixel intensities were calculated with background correction using ImageQuant software (v. 5.2, Molecular Dynamics, Piscataway, NJ).

For each sample, the slope of the regression line best fitting the linear range of the dilution curve was used to determine relative protein expression (Herrmann, P.C., *et al*. Mitochondrial proteome: altered cytochrome c oxidase subunit levels in prostate cancer. *Proteomics* **3**, 1801-10, 2003). Data were normalized to total protein and to the reference standard. The Wilcoxon rank-sum test was used to test group differences in the adjusted mean protein expression between normal and osteoarthritic chondrocytes. Two sided statistical tests are used throughout; P-values <0.05 were considered to be statistically significant. All statistical analyses were performed using the SSPS12.0 program. Linear regression analysis and graphing were performed by Origin 4.1. Hierarchical clustering was done using HCE 3.0 program (University of Maryland) with the approach adopted from cDNA microarrays.

**miRNA Target Prediction Methods.** The miRNA database miRBase (<http://microrna.sanger.ac.uk/>), the PicTar database (<http://pictar.bio.nyu.edu/>) and the TargetScan version 4.2 (<http://www.targetscan.org/index.html>) databases were used to identify potential miRNA targets. In order to have more accurate prediction results, we chose the targets genes that were predicted in two out of three databases and were conserved in other species.

**Ingenuity Pathway Analysis.** Ingenuity Pathways Analysis [IPA], (Ingenuity Systems, Mountain View, CA) is a curated database containing up-to-date information on over 20,000 mammalian genes and proteins, 1.4 million biological interactions, and 100 canonical pathways incorporating over 6,000 discreet gene concepts. This information is integrated with other relevant databases such as EntrezGene and Gene Ontology. The experimental datasets were used to query the IPA and to compose a set of interactive networks taking into consideration canonical pathways, the relevant biological interactions, and the cellular and disease processes.

**Primary cultures of human articular chondrocytes, normal and osteoarthritic.** Articular cartilage was transported from the surgical room in HBSS (Hanks Balanced Salt Solution) medium, was immediately dissected and subjected to sequential digestion with 1mg/ml pronase (Roche Applied Science, Germany) for 90 min and 1mg/ml collagenase P (Roche Applied Science, Germany) for 3 hours at 37^ο^C. Chondrocytes were counted and checked for viability using trypan blue staining. More than 95% of the cells were viable after isolation. Chondrocytes were then seeded in 6-well plates with Dulbecco’s Modified Eagles Medium/ Ham’s F-12 (DMEM/F-12) (GIBCO BRL, UK) plus 5% fetal bovine serum (FBS) and 100 U/ml penicillin-streptomycin and were incubated at 37^o^ C under a humidified 5% CO_2_ atmosphere.

**Real-time PCR analysis.** Transcription of 0.1μg RNA to cDNA was performed using the AMV Kit (Roche, Indianapolis, USA). LightCycler-FastStart DNA master SYBR Green, which contains Taq DNA polymerase, dNTP mix, SYBR Green I dye and MgCl_2_ (Roche, Indianapolis, USA) was used as a reaction mix for PCR. LightCycler Primer set (Roche, Indianapolis, USA) was used for the primers for GAPDH, as a housekeeping gene. Standard GAPDH cDNAs with known copy numbers were included in the LightCycler Primer set. Quantification was performed by real-time PCR (Light Cycler Instrument, Roche Molecular Systems, Alameda, CA) in 0.2 μl of cDNA for each analyzed sample using the LightCycler FastStart DNA Master HybProbe Kit (Roche, Penzberg, Germany) according to manufacturers’ instructions. The oligonucleotide primers used for LEP were 5’-TTCTTGTGGCTTTGGCCCTA-3’ and 5’GGAGACTGACTGCGTGTGTG

TGAA-3’, for MMP13 were 5'-CGCCAGAAGAATCTGTCTTTAAA-3', and 5'-CCAAATTATGGAGGAGATGC-3', for IL1B were 5’-CAACCAACAAGTGAT

ATTCTCCATG-3’ and 5’-GATCCACACTCTCCAGCTGCA-3’, for BMP7 were

5’-TGCCATCTCCGTCCTCTACT-3’ and 5’-GGGAAGGTCTCACAAAAGGCA

GTT-3’, for PPARA were 5’-AGAGCATGGTGCCTTCGCTGATGC-3’ and 5’-CAGTACATGTCTCTGTAGATCTCTTGC-3’, for ACAN were 5’-TCGAGGACA GCGAGGCC-3’ and 5’-TCGAGGGTGTAGGCGTGTAGAGA-3’. All samples were analyzed in triplicate and the average value of the triplicates was used for quantification. The data was expressed as the ratio of the levels of the target gene mRNA on that of the housekeeping gene GAPDH (gene mRNA copies/GAPDH copies), which was used as an internal control.

**Oligonucleotide transfections**. Chondrocytes were seeded in 6-well plates and were transfected with 50 nM siRNA against PPARA or BMP7 and 50nM of hsa-miR-22, hsa-miR-103, hsa-miR-29a and miR-22 inhibitor (Ambion Inc, TX, USA) using siPORT *NeoFX* transfection agent. SiPORT *NeoFX* is a lipid transfection agent consisting of a mixture of lipids that spontaneously complex small interference RNA and facilitates its transfer to the chondrocytes. Transfection with 50 nM scrambled siRNA or hsa-miR-negative control were used as a control. No cell toxicity was detected due to the transfection agent. RNA was extracted 24 and 48 hours after siRNA transfection and real-time PCR analysis was performed as described above. GAPDH levels were used as loading control. All oligonucleotide transfection experiments were performed in triplicate.

**Luciferase reporter assay.** Cells of 50% confluence in 24-well plates were transfected using Fugene6 (Roche, Penzberg, Germany). Firefly luciferase reporter gene constructs (200 ng) and 1 ng of the pRL-SV40 Renilla luciferase construct (for normalization) were cotransfected per well. Cell extracts were prepared 24–48 h after transfection, and the luciferase activity was measured using the Dual Luciferase Reporter Assay System (Promega, WI, USA).

**Protein extraction from chondrocytes.** OA and normal chondrocytes were trypsinised, collected and centrifuged for 10 min at 1700 rpm. The cell pellet was washed with PBS and then centrifuged as previously. The cell pellet was lysed using Nonidet P-40 lysis buffer containing 30 mM Tris (pH 7.5), 150 mM NaCl, 10% glycerol, 1% Nonidet P-40, and a cocktail of protease inhibitors for 30 min on ice, followed by centrifugation for 15 min at 12,000 rpm. The supernatant was transferred in another tube and stored at -20^o^C. Protein concentration was quantified using the Bio-Rad Bradford protein assay (Bio-Rad, Hercules, CA) with bovine serum albumen as standard.

**Western blot analysis and quantification.** Cell lysates from normal and OA chondrocytes were electrophoresed and separated on a 4-20% Tris-HCl gel (Bio-Rad, Hercules, CA) and transferred to a Hybond-ECL nitrocellulose membrane (Amersham Biosciences, Piscataway, NJ). The membrane was probed with antibodies described in suppl. Table 3. The nitrocellulose membranes were then exposed to photographic film, which was scanned and the intensities of the protein bands, which were expressed as arbitrary units, were determined by computerized densitometry. Protein levels were normalized to β-actin protein levels. Protein expression levels of Western blots were quantified using the Image Quant 6 program analysis.

**Enzyme-linked immunosorbent assay (ELISA).** Concentrations of MMP-13 in cell culture supernatants were evaluated by ELISA (Biotrak ELISA kit, RPN2621, Amersham). The limits of detection of these kits (mean ± SD) were 0.094 ± 0.03 ng/ml for MMP-13. Chondrocyte conditioned media were collected and centrifuged for 10 minutes at 10,000 rpm to remove cellular debris.

**Immunofluorescence.** Cells were seeded on fibronectin-coated cell culture slides (Becton Dickinson, NJ, USA), fixed for 10 min in 3.7% PBS-buffered formaldehyde, blocked with 3% BSA, and incubated with primary antisera for MMP-13 (1:500 dilution) (H230, Santa Cruz Biotech Inc, CA, USA), for 2 h in 1% BSA in PBS, and with secondary antisera under the same conditions. Secondary sera used were anti-rabbit FITC-conjugated anti-IgG (Santa Cruz Biotech Inc, CA, USA).
