# Supplementary material for: Integrative MicroRNA and Proteomic Approaches Identify Novel Osteoarthritis Genes and Their Collaborative Metabolic and Inflammatory Networks
Source: PLoS One. 2008 Nov 17;3(11):e3740. doi: 10.1371/journal.pone.0003740 (PMC2582945; doi:10.1371/journal.pone.0003740)
Supplement: Table S1 — (0.07 MB PPT) [file pone.0003740.s002.ppt]

## Slide 1
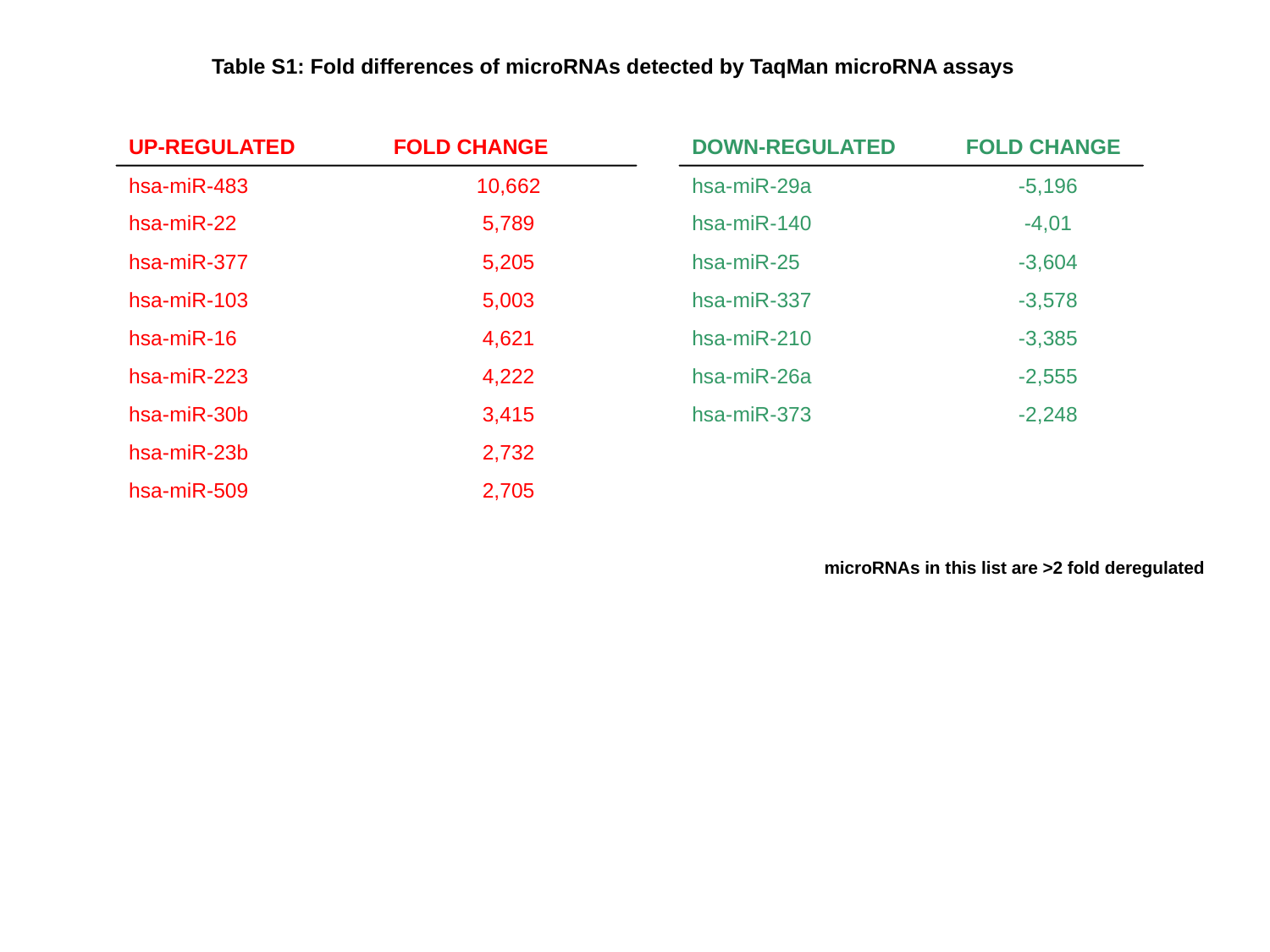

Table S1: Fold differences of microRNAs detected by TaqMan microRNA assays
UP-REGULATED
FOLD CHANGE
DOWN-REGULATED
FOLD CHANGE
hsa-miR-483
10,662
hsa-miR-29a
-5,196
hsa-miR-22
5,789
hsa-miR-140
-4,01
hsa-miR-377
5,205
hsa-miR-25
-3,604
hsa-miR-103
5,003
hsa-miR-337
-3,578
hsa-miR-16
4,621
hsa-miR-210
-3,385
hsa-miR-223
4,222
hsa-miR-26a
-2,555
hsa-miR-30b
3,415
hsa-miR-373
-2,248
hsa-miR-23b
2,732
hsa-miR-509
2,705
microRNAs in this list are >2 fold deregulated
