# Supplementary material for: Integrative MicroRNA and Proteomic Approaches Identify Novel Osteoarthritis Genes and Their Collaborative Metabolic and Inflammatory Networks
Source: PLoS One. 2008 Nov 17;3(11):e3740. doi: 10.1371/journal.pone.0003740 (PMC2582945; doi:10.1371/journal.pone.0003740)
Supplement: Table S2 — (0.07 MB PPT) [file pone.0003740.s003.ppt]

## Slide 1
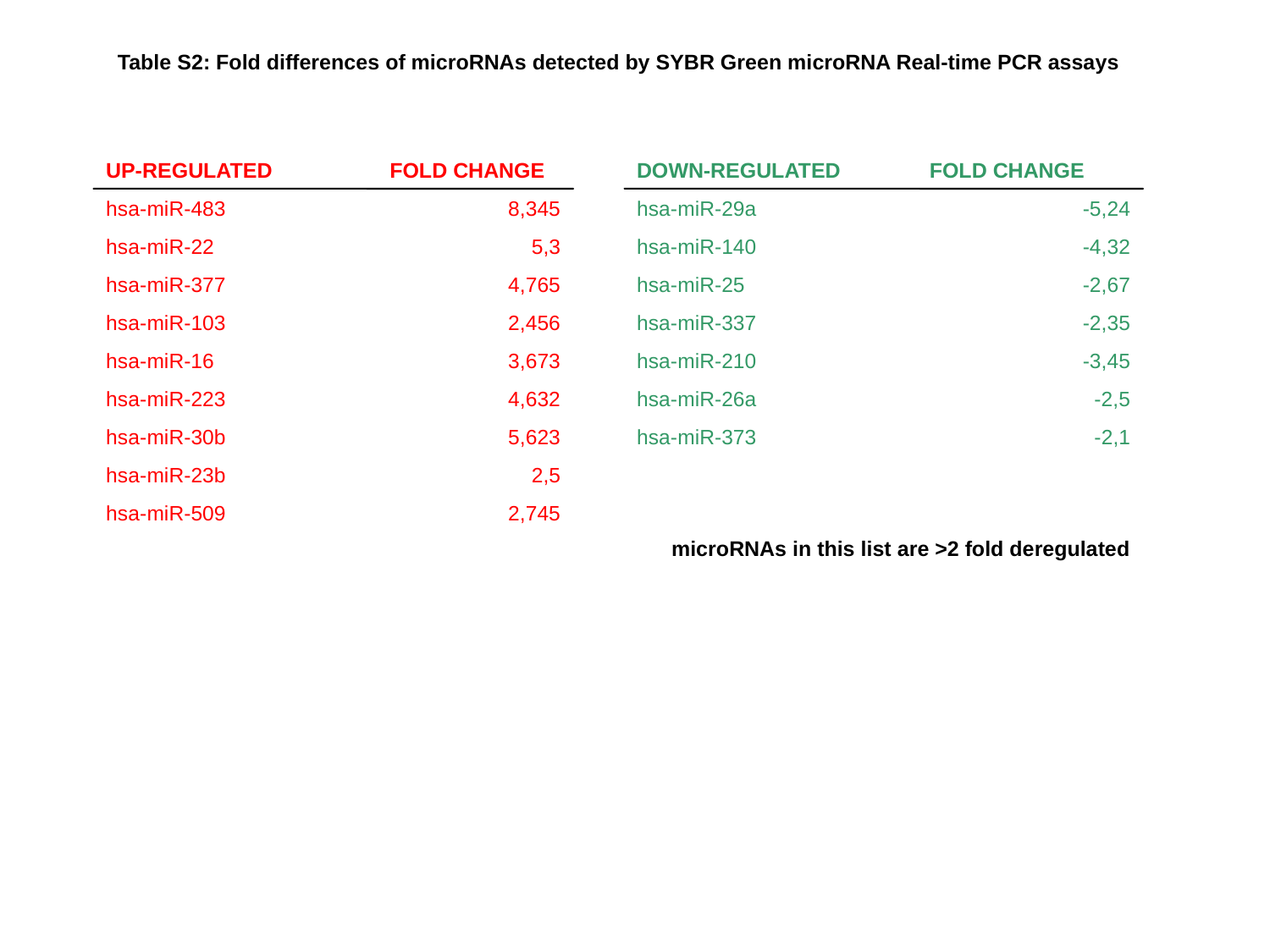

Table S2: Fold differences of microRNAs detected by SYBR Green microRNA Real-time PCR assays
UP-REGULATED
FOLD CHANGE
DOWN-REGULATED
FOLD CHANGE
hsa-miR-483
8,345
hsa-miR-29a
-5,24
hsa-miR-22
5,3
hsa-miR-140
-4,32
hsa-miR-377
4,765
hsa-miR-25
-2,67
hsa-miR-103
2,456
hsa-miR-337
-2,35
hsa-miR-16
3,673
hsa-miR-210
-3,45
hsa-miR-223
4,632
hsa-miR-26a
-2,5
hsa-miR-30b
5,623
hsa-miR-373
-2,1
hsa-miR-23b
2,5
hsa-miR-509
2,745
microRNAs in this list are >2 fold deregulated
