# Supplementary figures and images for: Integrative MicroRNA and Proteomic Approaches Identify Novel Osteoarthritis Genes and Their Collaborative Metabolic and Inflammatory Networks
Source: PLoS One. 2008 Nov 17;3(11):e3740. doi: 10.1371/journal.pone.0003740 (PMC2582945; doi:10.1371/journal.pone.0003740)

## Slide 1
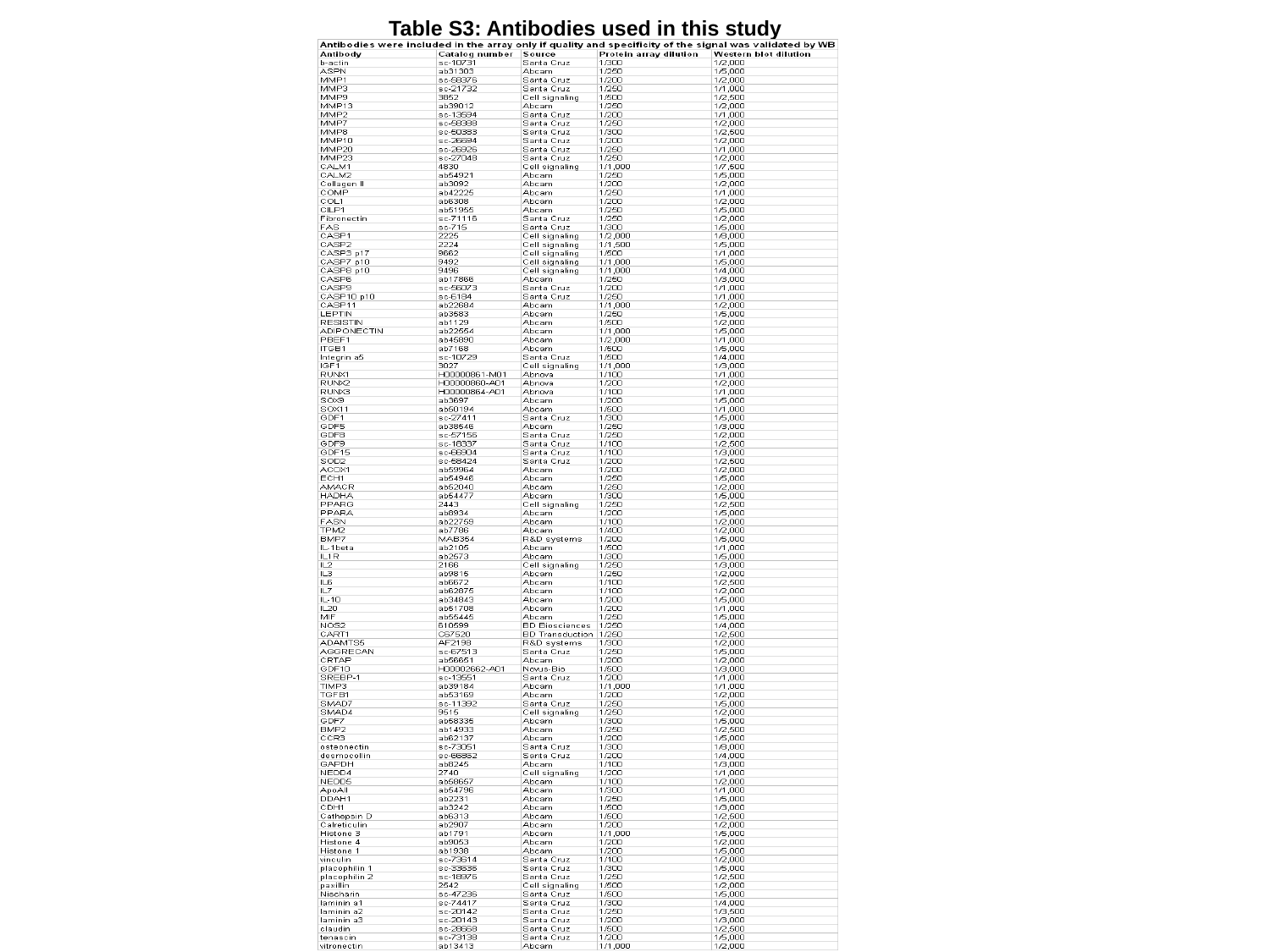

Table S3: Antibodies used in this study

## Slide 2
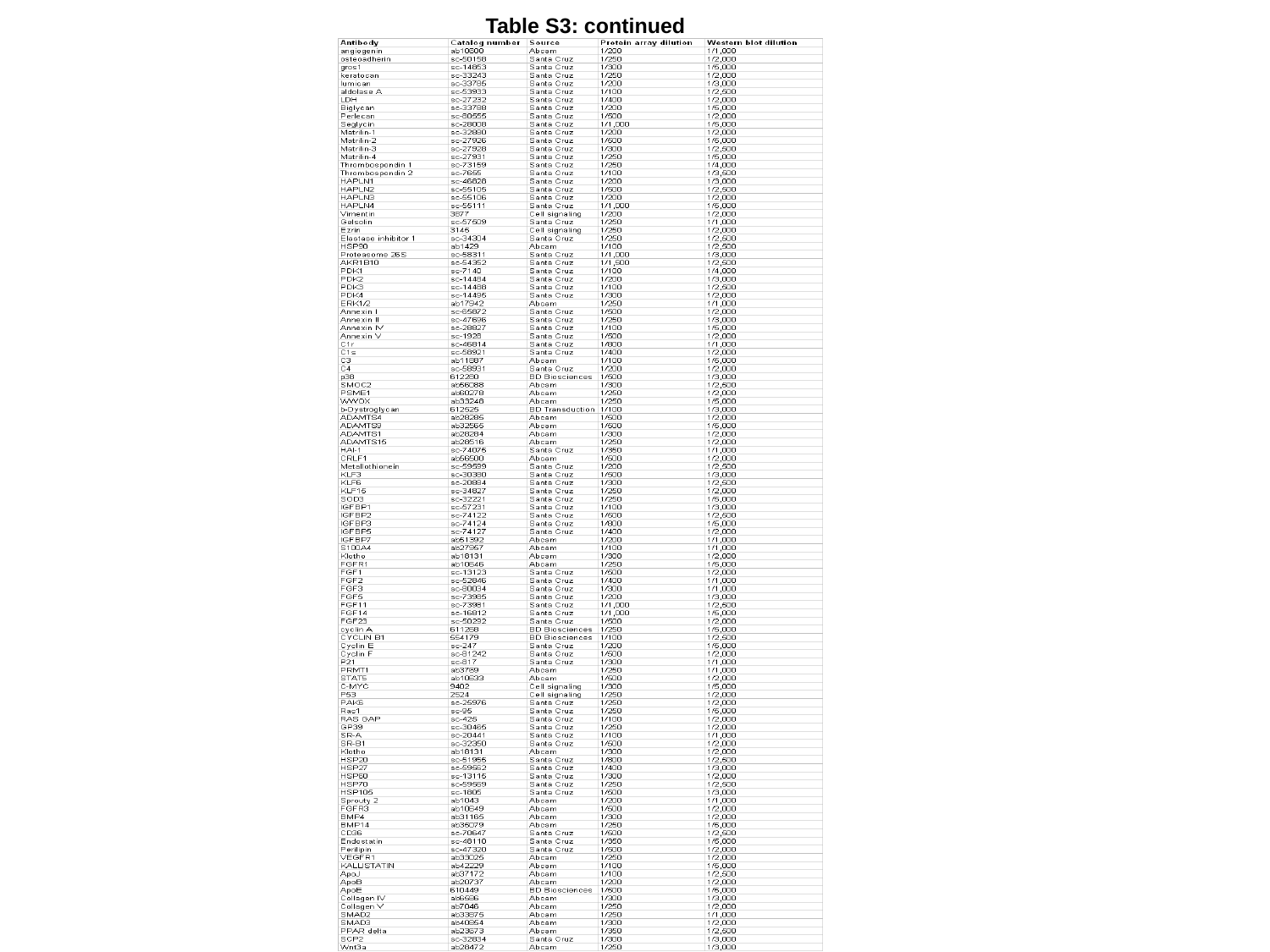

Table S3: continued

Supplement: Table S3 — (0.13 MB PPT) [file pone.0003740.s004.ppt]
