# Supplementary material for: Integrative MicroRNA and Proteomic Approaches Identify Novel Osteoarthritis Genes and Their Collaborative Metabolic and Inflammatory Networks
Source: PLoS One. 2008 Nov 17;3(11):e3740. doi: 10.1371/journal.pone.0003740 (PMC2582945; doi:10.1371/journal.pone.0003740)
Supplement: Table S4 — (0.07 MB PPT) [file pone.0003740.s005.ppt]

## Slide 1
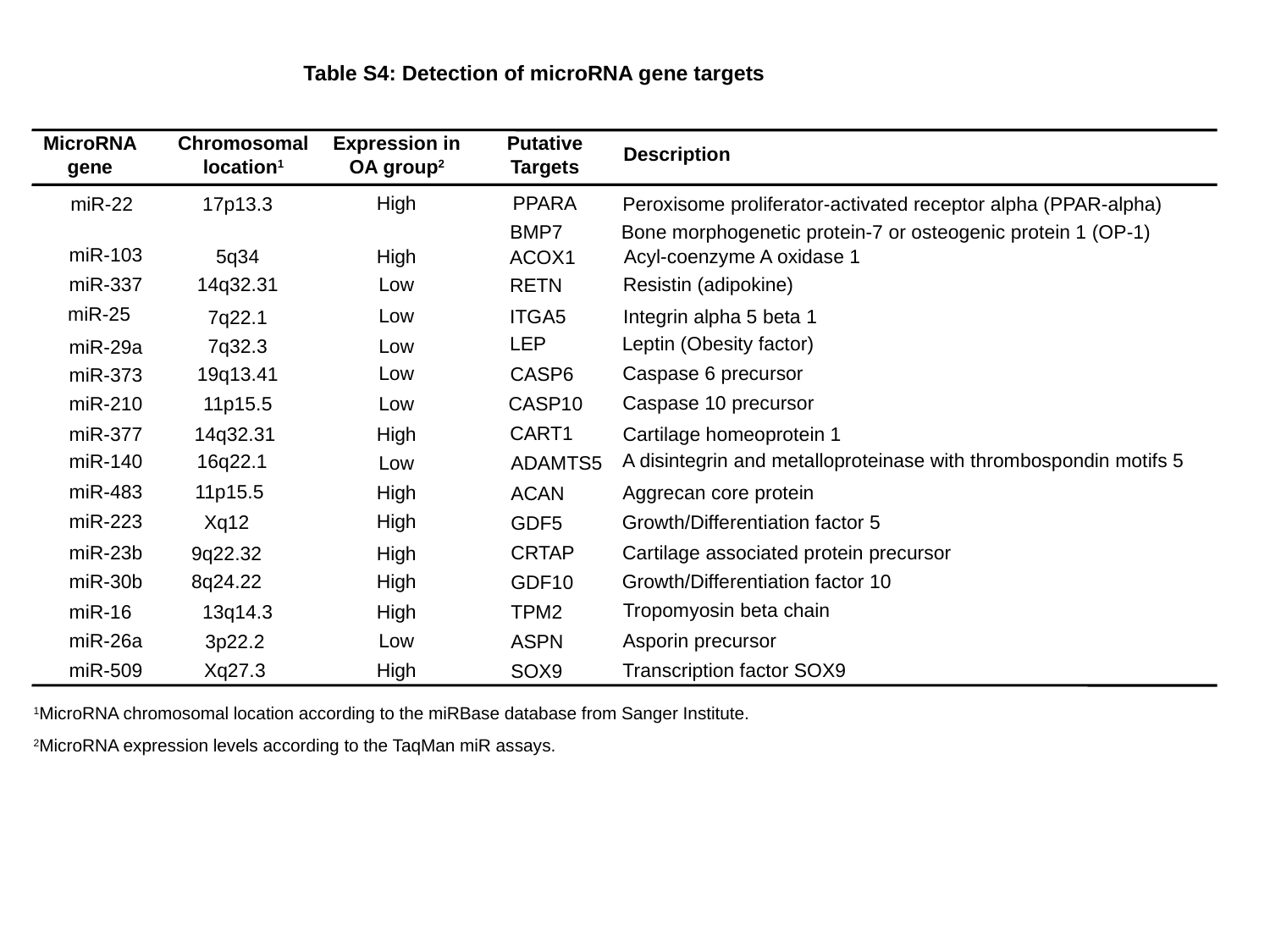

Table S4: Detection of microRNA gene targets
MicroRNA gene
Chromosomal location1
Expression in OA group2
Putative Targets
Description
High
PPARA
Peroxisome proliferator-activated receptor alpha (PPAR-alpha)
miR-22
17p13.3
BMP7
Bone morphogenetic protein-7 or osteogenic protein 1 (OP-1)
miR-103
5q34
High
Acyl-coenzyme A oxidase 1
ACOX1
Low
miR-337
14q32.31
Resistin (adipokine)
RETN
miR-25
Low
Integrin alpha 5 beta 1
ITGA5
7q22.1
Leptin (Obesity factor)
LEP
7q32.3
Low
miR-29a
Low
Caspase 6 precursor
CASP6
19q13.41
miR-373
Caspase 10 precursor
Low
miR-210
11p15.5
CASP10
CART1
High
miR-377
14q32.31
Cartilage homeoprotein 1
A disintegrin and metalloproteinase with thrombospondin motifs 5
miR-140
16q22.1
Low
ADAMTS5
miR-483
11p15.5
Aggrecan core protein
High
ACAN
miR-223
High
Xq12
Growth/Differentiation factor 5
GDF5
miR-23b
CRTAP
Cartilage associated protein precursor
High
9q22.32
miR-30b
8q24.22
High
Growth/Differentiation factor 10
GDF10
Tropomyosin beta chain
miR-16
13q14.3
High
TPM2
miR-26a
Low
Asporin precursor
3p22.2
ASPN
miR-509
Xq27.3
High
Transcription factor SOX9
SOX9
1MicroRNA chromosomal location according to the miRBase database from Sanger Institute.
2MicroRNA expression levels according to the TaqMan miR assays.
