# Supplementary material for: Integrative MicroRNA and Proteomic Approaches Identify Novel Osteoarthritis Genes and Their Collaborative Metabolic and Inflammatory Networks
Source: PLoS One. 2008 Nov 17;3(11):e3740. doi: 10.1371/journal.pone.0003740 (PMC2582945; doi:10.1371/journal.pone.0003740)
Supplement: Figure S1 — (0.16 MB PPT) [file pone.0003740.s006.ppt]

## Slide 1
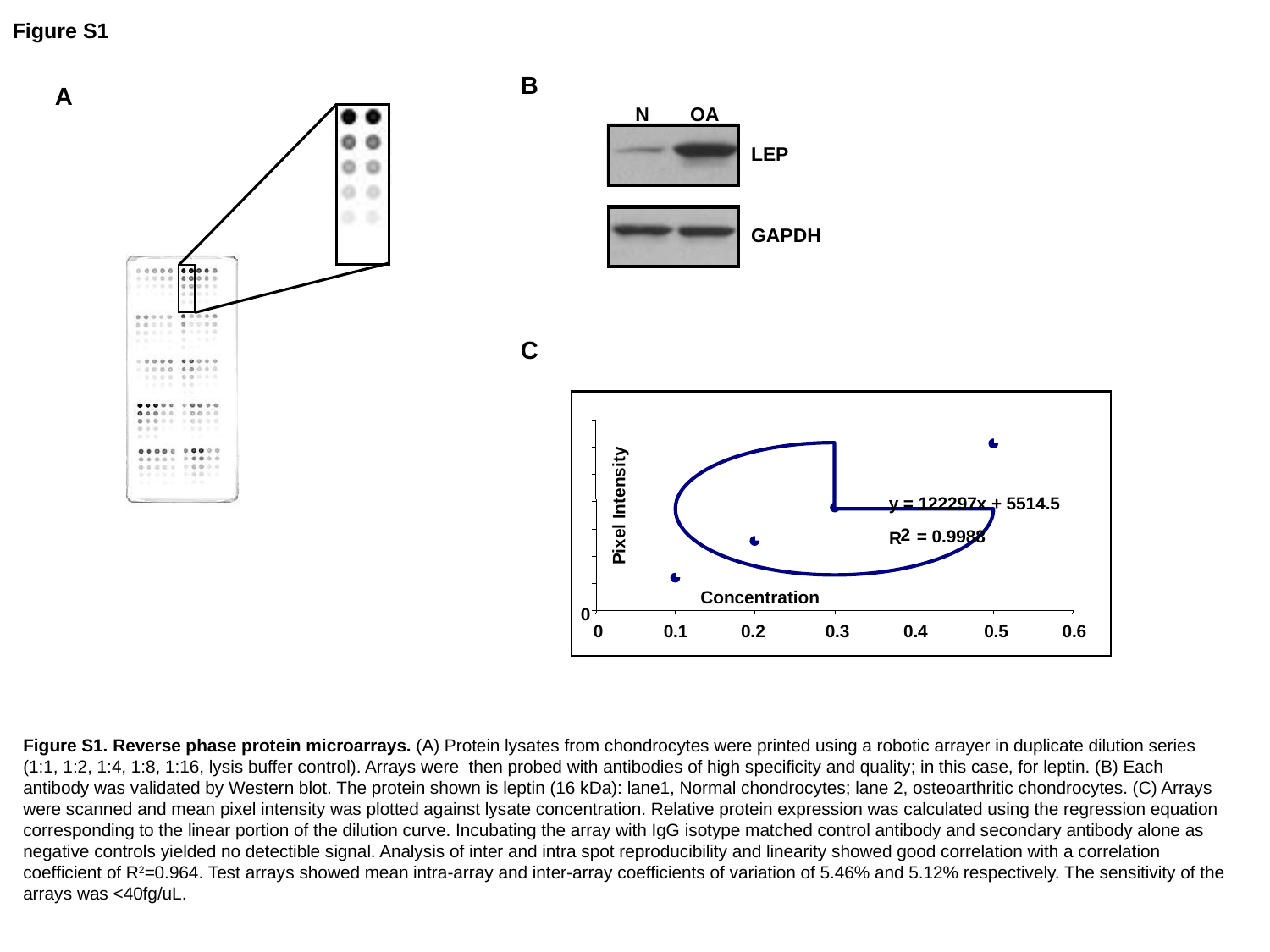

Figure S1
B
A
N
OA
LEP
GAPDH
C
Pixel Intensity
y = 122297x + 5514.5
2
 = 0.9988
R
Concentration
0
0
0.1
0.2
0.3
0.4
0.5
0.6
Figure S1. Reverse phase protein microarrays. (A) Protein lysates from chondrocytes were printed using a robotic arrayer in duplicate dilution series (1:1, 1:2, 1:4, 1:8, 1:16, lysis buffer control). Arrays were then probed with antibodies of high specificity and quality; in this case, for leptin. (B) Each antibody was validated by Western blot. The protein shown is leptin (16 kDa): lane1, Normal chondrocytes; lane 2, osteoarthritic chondrocytes. (C) Arrays were scanned and mean pixel intensity was plotted against lysate concentration. Relative protein expression was calculated using the regression equation corresponding to the linear portion of the dilution curve. Incubating the array with IgG isotype matched control antibody and secondary antibody alone as negative controls yielded no detectible signal. Analysis of inter and intra spot reproducibility and linearity showed good correlation with a correlation coefficient of R2=0.964. Test arrays showed mean intra-array and inter-array coefficients of variation of 5.46% and 5.12% respectively. The sensitivity of the arrays was <40fg/uL.
