# Supplementary material for: Integrative MicroRNA and Proteomic Approaches Identify Novel Osteoarthritis Genes and Their Collaborative Metabolic and Inflammatory Networks
Source: PLoS One. 2008 Nov 17;3(11):e3740. doi: 10.1371/journal.pone.0003740 (PMC2582945; doi:10.1371/journal.pone.0003740)
Supplement: Figure S2 — (0.12 MB PPT) [file pone.0003740.s007.ppt]

## Slide 1
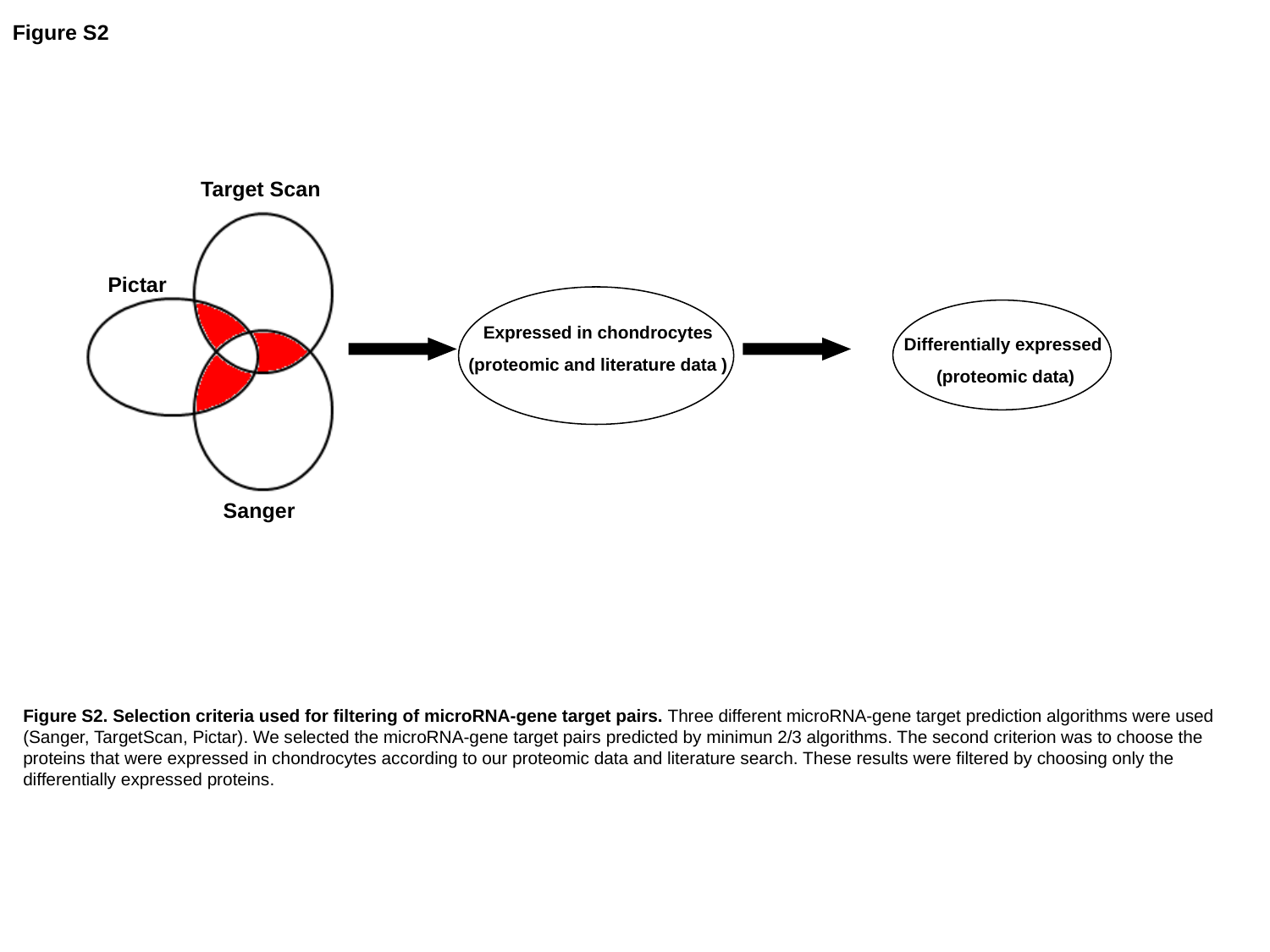

Figure S2
Target Scan
Pictar
Expressed in chondrocytes
(proteomic and literature data )
Differentially expressed
(proteomic data)
Sanger
Figure S2. Selection criteria used for filtering of microRNA-gene target pairs. Three different microRNA-gene target prediction algorithms were used (Sanger, TargetScan, Pictar). We selected the microRNA-gene target pairs predicted by minimun 2/3 algorithms. The second criterion was to choose the proteins that were expressed in chondrocytes according to our proteomic data and literature search. These results were filtered by choosing only the differentially expressed proteins.
