# Supplementary material for: Integrative MicroRNA and Proteomic Approaches Identify Novel Osteoarthritis Genes and Their Collaborative Metabolic and Inflammatory Networks
Source: PLoS One. 2008 Nov 17;3(11):e3740. doi: 10.1371/journal.pone.0003740 (PMC2582945; doi:10.1371/journal.pone.0003740)
Supplement: Figure S3 — (0.68 MB PPT) [file pone.0003740.s008.ppt]

## Slide 1
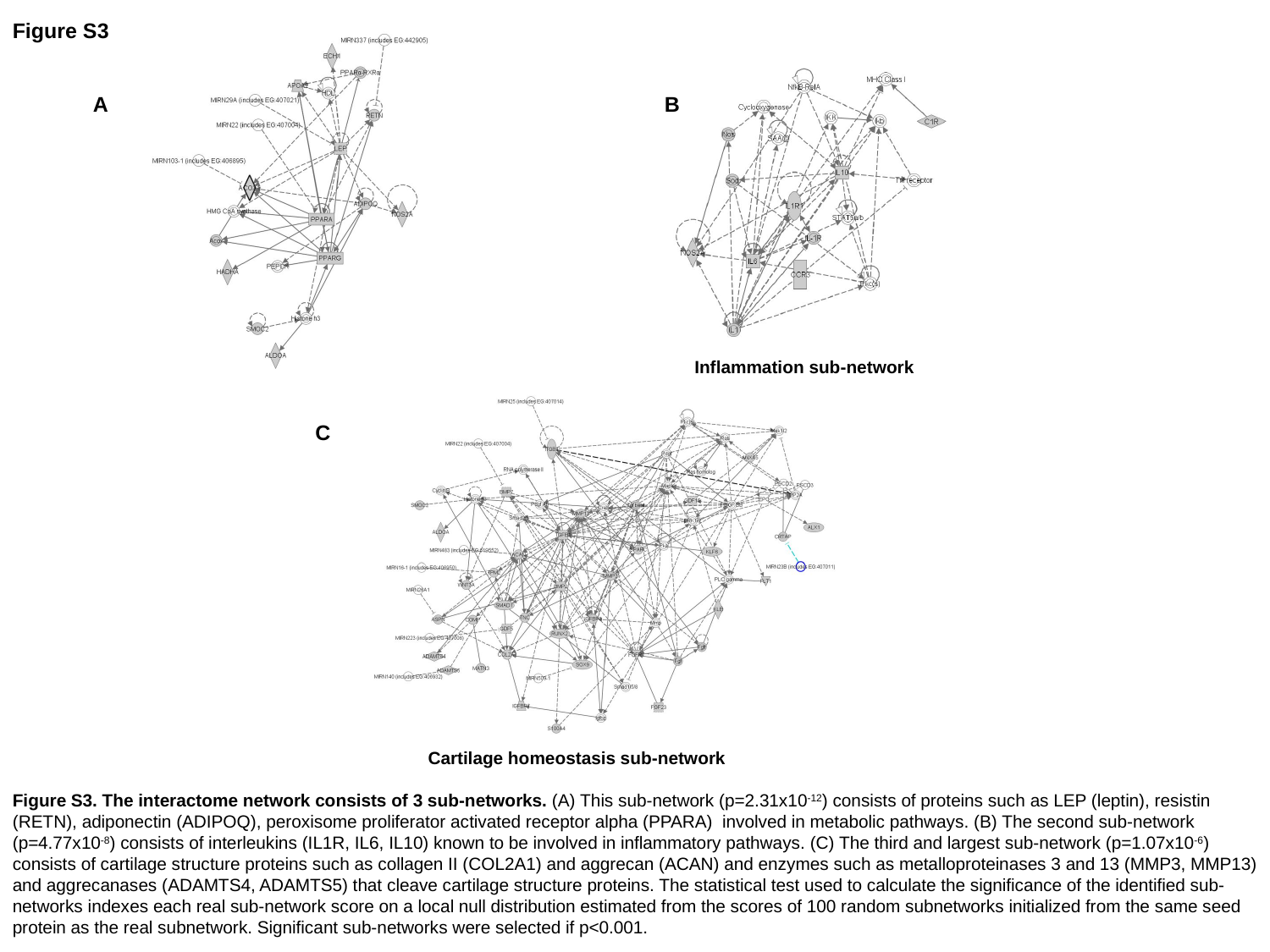

Figure S3
A
B
Inflammation sub-network
Metabolism sub-network
C
Cartilage homeostasis sub-network
Figure S3. The interactome network consists of 3 sub-networks. (A) This sub-network (p=2.31x10^-12^) consists of proteins such as LEP (leptin), resistin (RETN), adiponectin (ADIPOQ), peroxisome proliferator activated receptor alpha (PPARA) involved in metabolic pathways. (B) The second sub-network (p=4.77x10^-8^) consists of interleukins (IL1R, IL6, IL10) known to be involved in inflammatory pathways. (C) The third and largest sub-network (p=1.07x10^-6^) consists of cartilage structure proteins such as collagen II (COL2A1) and aggrecan (ACAN) and enzymes such as metalloproteinases 3 and 13 (MMP3, MMP13) and aggrecanases (ADAMTS4, ADAMTS5) that cleave cartilage structure proteins. The statistical test used to calculate the significance of the identified sub-networks indexes each real sub-network score on a local null distribution estimated from the scores of 100 random subnetworks initialized from the same seed protein as the real subnetwork. Significant sub-networks were selected if p<0.001.
