# Supplementary material for: Integrative MicroRNA and Proteomic Approaches Identify Novel Osteoarthritis Genes and Their Collaborative Metabolic and Inflammatory Networks
Source: PLoS One. 2008 Nov 17;3(11):e3740. doi: 10.1371/journal.pone.0003740 (PMC2582945; doi:10.1371/journal.pone.0003740)
Supplement: Figure S4 — (0.14 MB PPT) [file pone.0003740.s009.ppt]

## Slide 1
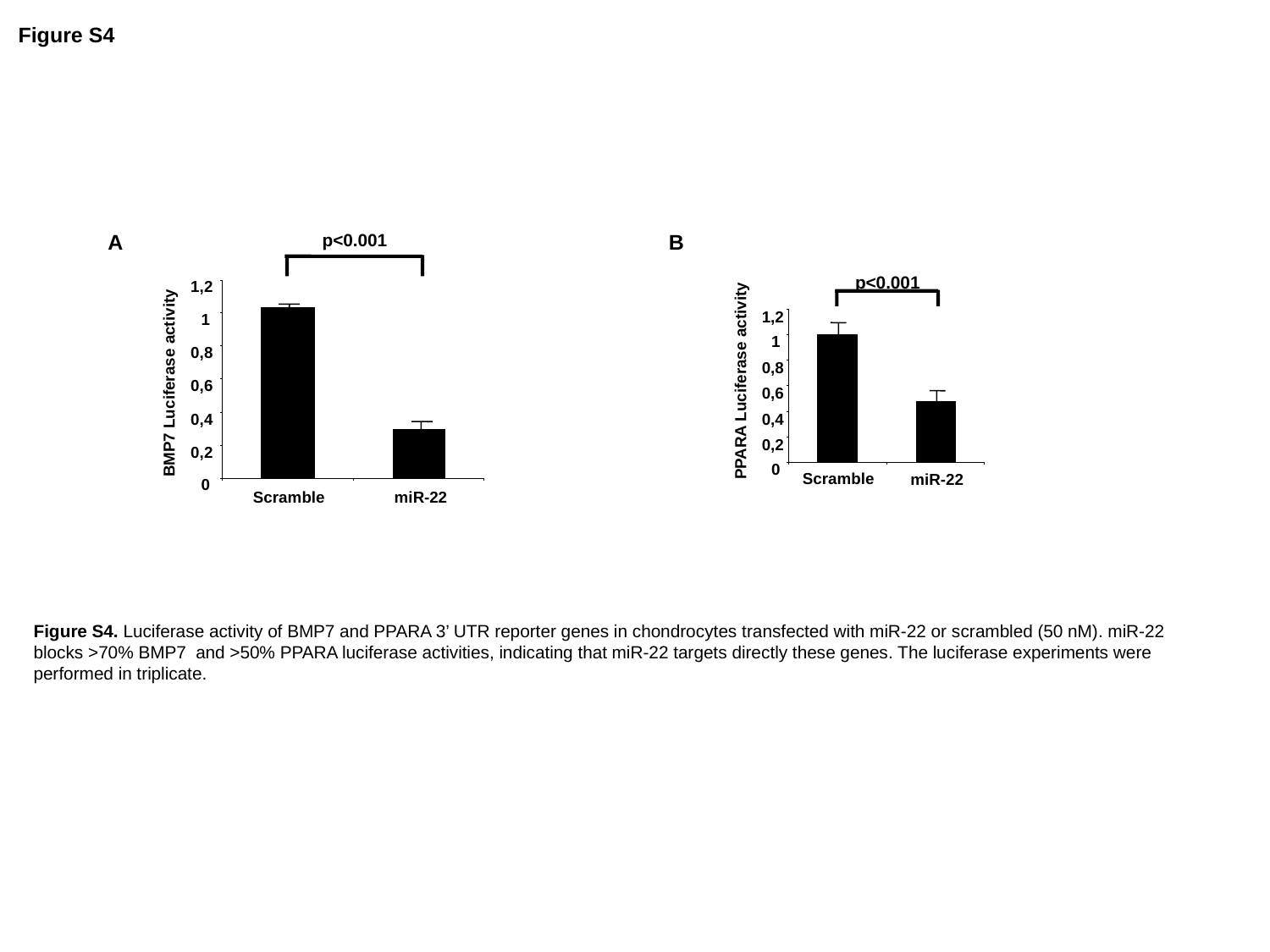

Figure S4
A
p<0.001
1,2
1
0,8
BMP7 Luciferase activity
0,6
0,4
0,2
0
Scramble
miR-22
B
p<0.001
1,2
1
0,8
PPARA Luciferase activity
0,6
0,4
0,2
0
Scramble
miR-22
Figure S4. Luciferase activity of BMP7 and PPARA 3’ UTR reporter genes in chondrocytes transfected with miR-22 or scrambled (50 nM). miR-22 blocks >70% BMP7 and >50% PPARA luciferase activities, indicating that miR-22 targets directly these genes. The luciferase experiments were performed in triplicate.
