# Supplementary material for: Integrative MicroRNA and Proteomic Approaches Identify Novel Osteoarthritis Genes and Their Collaborative Metabolic and Inflammatory Networks
Source: PLoS One. 2008 Nov 17;3(11):e3740. doi: 10.1371/journal.pone.0003740 (PMC2582945; doi:10.1371/journal.pone.0003740)
Supplement: Figure S5 — (0.14 MB PPT) [file pone.0003740.s010.ppt]

## Slide 1
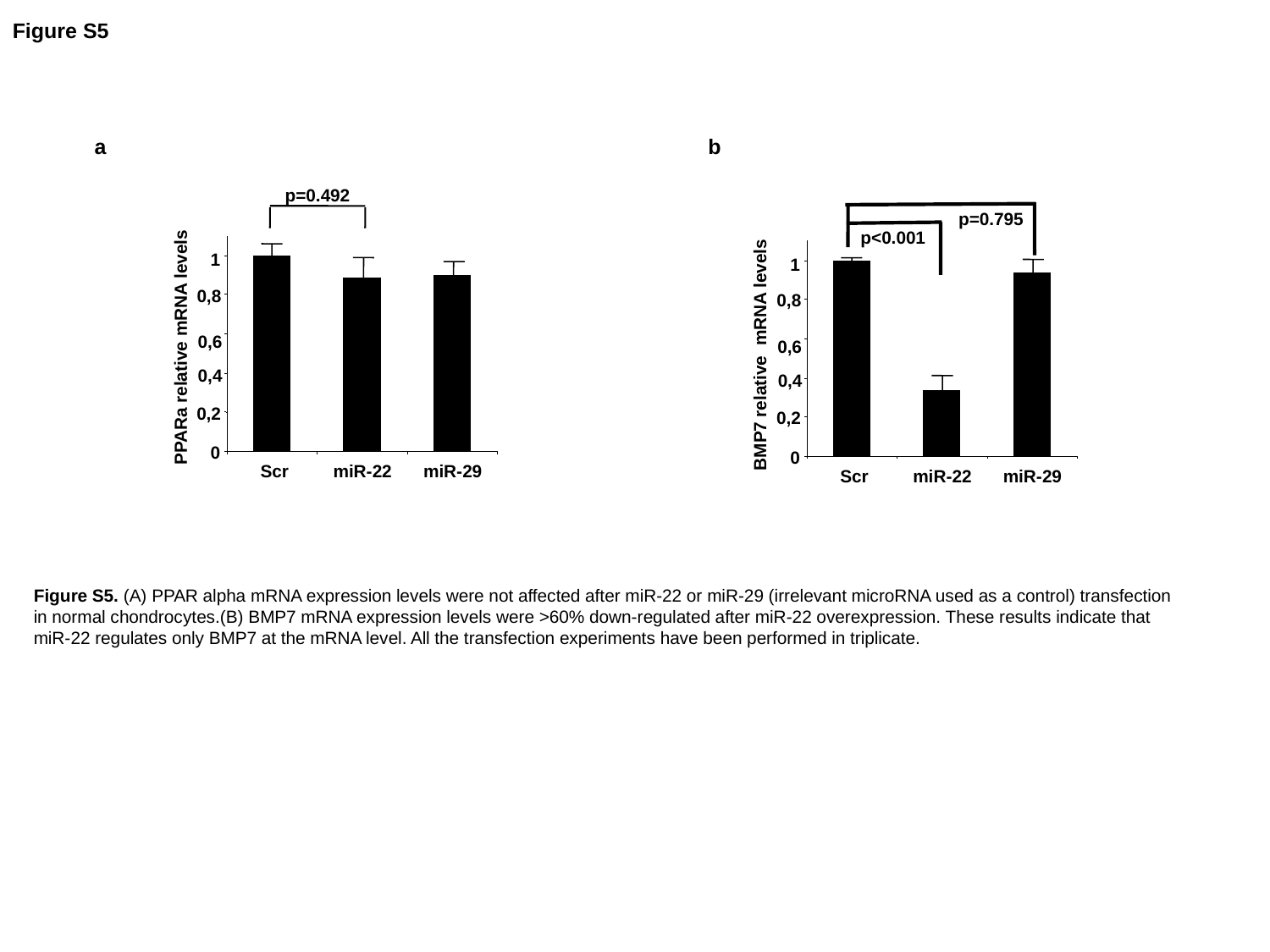

Figure S5
a
b
p=0.492
1
0,8
0,6
PPARa relative mRNA levels
0,4
0,2
0
Scr
miR-22
miR-29
p=0.795
p<0.001
1
0,8
0,6
BMP7 relative mRNA levels
0,4
0,2
0
Scr
miR-22
miR-29
Figure S5. (A) PPAR alpha mRNA expression levels were not affected after miR-22 or miR-29 (irrelevant microRNA used as a control) transfection in normal chondrocytes.(B) BMP7 mRNA expression levels were >60% down-regulated after miR-22 overexpression. These results indicate that miR-22 regulates only BMP7 at the mRNA level. All the transfection experiments have been performed in triplicate.
